# Comparative Analysis of Stromal Vascular Fraction and Alternative Mechanisms in Bone Fracture Stimulation to Bridge the Gap between Nature and Technological Advancement: A Systematic Review

**DOI:** 10.3390/biomedicines12020342

**Published:** 2024-02-01

**Authors:** Evgeniy Nikolaevich Goncharov, Oleg Aleksandrovich Koval, Eduard Nikolaevich Bezuglov, Mikhail Engelgard, Eremin Ilya Igorevich, Konstantin Velentinovich Kotenko, Manuel De Jesus Encarnacion Ramirez, Nicola Montemurro

**Affiliations:** 1 Petrovsky Russian Scientific Center of Surgery, 121359 Moscow, Russia; 2 Department of Neurosurgery, Peoples’ Friendship University of Russia (RUDN University), 117198 Moscow, Russia; 3 Department of Neurosurgery, Azienda Ospedaliero Universitaria Pisana (AOUP), 56100 Pisa, Italy

**Keywords:** regenerative medicine, stromal vascular fraction, tissue regeneration, graft survival, surgery

## Abstract

Background: Various stimulation methods, including electrical, ultrasound, mechanical, and biological interventions, are explored, each leveraging intricate cellular and molecular dynamics to expedite healing. The advent of stromal vascular fraction (SVF) marks a significant stride, offering multifarious benefits in bone healing, from enhanced bone formation to optimal vascular integration, drawing a harmonious balance between innate mechanisms and scientific advancements. Methods: This systematic review was conducted focusing on literature from 2016 to 2023 and encompassing various bone healing stimulation mechanisms like SVF, electrical, ultrasound, and mechanical stimulation. The extracted data underwent meticulous synthesis and analysis, emphasizing comparative evaluations of mechanisms, applications, and outcomes of each intervention. Results: The reviewed studies reveal the potential of SVF in bone fracture healing, with its regenerative and anti-inflammatory effects. The purification of SVF is crucial for safe therapeutic use. Characterization involves flow cytometry and microscopy. Studies show SVF’s efficacy in bone regeneration, versatility in various contexts, and potential for clinical use. SVF appears superior to electrical, ultrasound, and mechanical stimulation, with low complications. Conclusions: This review compares bone healing methods, including SVF. It provides valuable insights into SVF’s potential for bone regeneration. However, due to limited human studies and potential bias, cautious interpretation is necessary. Further research is essential to validate these findings and determine the optimal SVF applications in bone healing.

## 1. Introduction

The healing of fractures, such as a broken femur, indicated the presence of caregiving in early civilizations, a concept highlighted by anthropologist Margaret Mead [1,2]. Over time, the approach to treating bone fractures evolved from simple realignment to enhancing and speeding up the healing process [1]. This evolution included the use of herbal remedies in ancient times, the introduction of plaster casts in the 19th century, and modern technological advancements. Bone healing itself is a complex, naturally orchestrated process involving several phases of cellular and molecular activity, showcasing the body’s remarkable ability to repair and regenerate [3,4]. The process is generated by a fracture, setting off an acute inflammatory response. At the molecular level, cytokines and chemokines are rapidly released, acting as beacon signals for immune cells like neutrophils and macrophages to merge on the injury site [5]. Concurrently, the activation of the blood clotting cascade forms a hematoma at the fracture site, providing a provisional matrix for incoming cells. This initial phase, while protective, also diligently prepares the groundwork for the healing stages that lie ahead [5,6].

Transitioning from inflammation, the reparative phase is ushered in. Central to this phase are chondrocytes, fibroblasts, and osteoblasts. Chondrocytes initiate the cartilage formation, laying the groundwork for subsequent bone formation. Fibroblasts, driven by growth factors like bone morphogenetic proteins (BMPs), lay down a collagen-rich matrix, culminating in a soft callus that encases the fracture. This platform is transient, serving as a placeholder until osteoblasts take the lead. Osteoblasts initiate the synthesis of new bone tissue, gradually transforming the soft callus into a sturdier bony callus [7].

The process culminates with the remodeling phase, where osteoclasts—cells adept at bone resorption—begin their meticulous task. With the interplay of molecular signals and growth factors, osteoclasts fine-tune the newly formed bone, ensuring its structure and strength are harmoniously aligned with the original bone. Simultaneously, osteoblasts continue to deposit bone, achieving a delicate balance between bone formation and resorption [8].

This knowledge has not only revealed the marvels of nature but has also laid a solid foundation, paving the way for the development of numerous techniques designed to amplify and stimulate this innate process. At the vanguard is electrical stimulation, often described as a process between cellular dynamics and electrical currents [8,9]. Electrical stimulation can trigger changes in the concentrations of ions within the cell, which can in turn activate or inhibit specific signaling pathways [10]. This can lead to alterations in gene expression, protein synthesis, and cellular metabolism. Furthermore, electrical stimulation has been shown to promote cell proliferation, differentiation, and migration [11]. For example, in the field of regenerative medicine, electrical stimulation has been used to enhance the healing of tissues by promoting the growth and differentiation of stem cells. In addition, electrical stimulation can affect the release of various signaling molecules, such as neurotransmitters and growth factors, which can influence neighboring cells and tissue migration [10,11]. For instance, direct current stimulation necessitates a surgical insertion of electrodes directly at the fracture’s epicenter. Once in place, these electrodes discharge a continuous electric current, which vitalizes cellular processes pivotal to bone repair. A less invasive sibling to this approach is capacitive coupling stimulation, employing external electrodes to conjure an electric field that envelops and nurtures the fracture from the outside, mitigating the need for any internal implants. It would be noteworthy to mention that DCS is not widely used, and CCS has limited efficacy to certain types of fractures. Another player in this field is pulsed electromagnetic field (PEMF) therapy. Here, an external apparatus crafts a distinct electromagnetic field, serving as a catalyst that invigorates cells and essential biochemical reactions, steering the path of bone regeneration [9,10,11].

Next, we have ultrasound stimulation, where the very vibrations of sound waves become the healers. Specific frequencies and intensities of these waves have been shown to have a compelling influence on cellular activities. A prime technique, low-intensity pulsed ultrasound (LIPUS), focuses these waves on the fracture, inducing a surge in osteoblast activities, the main architects of bone, accelerating the healing trajectory [11,12].

Mechanical stimulation utilizes specialized devices to apply measured forces onto the fracture, rekindling the cellular dance of bone formation. Additionally, vibration therapy, with its precision-calibrated shakes, has proven to be a boon, especially for those grappling with conditions like osteoporosis, fortifying weakened bones [13]. In the 19th century, the German anatomist Julius Wolff proposed “Wolff’s Law”, stating that bone remodels in response to the forces or stresses placed upon it. This principle emphasizes that bone density and architecture can change based on the functional forces it experiences. Therefore, areas subjected to more significant loads become stronger, whereas those with less mechanical stimulation weaken over time [14].

Dynamic mechanical stimulation involves exerting controlled forces or loads directly on the fracture site, prompting the cellular mechanisms responsible for bone formation. By mimicking the natural stresses bone would typically experience, this method aims to accelerate the healing process [15].

Vibration therapy is an alternative to direct mechanical loading; vibration therapy exposes the entire skeleton or specific parts to low-magnitude, high-frequency mechanical stimuli. When mechanical forces are applied to bone, osteocytes sense this change and release signaling molecules. Low-intensity vibration (LIV), (35–90 Hz, 15 min/daily) resulting in improved callus density, enlarged callus area and width, accelerated osteotomy bridging, upregulated osteocalcin expression, and suppressed osteoclast activity at 30 days [11,16]. This cellular communication often results in the recruitment of bone-forming cells, osteoblasts, and the inhibition of bone-resorbing cells, or osteoclasts [17]. BMPs belong to the transforming growth factor-beta (TGF-β) superfamily. Several BMPs have been studied for their role in bone healing, with BMP-2 and BMP-7 (also known as osteogenic protein-1 or OP-1) being the most extensively researched and clinically applied, accelerating bone growth when introduced to the fracture zone. BMPs significantly improved healing rates in long bone nonunion and were a viable alternative to autografts [4,11,18]. Then, there is the platelet-rich plasma (PRP), a concentrate rich in growth factors, derived directly from the patient, acting as a potent agent for regeneration. Not to forget the potential benefits of stem cell therapy, wherein the chameleon-like mesenchymal stem cells, with their ability to transform into bone cells, bring significant reinforcements to the healing front [5,19].

Nutritional and pharmacological stimulation contributes by providing nutrients and support to enhance the bone’s healing process. Essential elements like vitamin D and calcium stand as cornerstones of bone health, ensuring a strong foundation for fracture healing. Medications such as bisphosphonates, beyond their traditional role in osteoporosis treatment, assist in the recovery process by reducing bone decomposition. Meanwhile, the periodic administration of the parathyroid hormone (PTH) acts as a regenerative catalyst, amplifying bone repair mechanisms [5,20].

SVF is a heterogeneous mixture of cells, pericytes, smooth muscle cells, and adipose-derived stem cells (ADSCs) [21]. These cells play a crucial role in tissue regeneration and repair, primarily due to their ability to differentiate into various cell types and release angiogenic and anti-inflammatory factors [22]. Among these, the ADSCs are particularly notable for their multipotency, enabling them to differentiate into various cell types, such as adipocytes, osteoblasts, and chondrocytes, under appropriate conditions [21]. Moreover, these cells are known for their angiogenic and immunomodulatory capabilities, primarily due to their secretion of growth factors and cytokines [23]. While the regenerative and reparative capacities can be traced back to the ADSCs, the immune cells within SVF contribute to the immunomodulatory effects, essential for tissue repair and regeneration [24]. Over the following decades, this recognition spiraled into a flurry of research investigating the regenerative potential of SVF, spurred by its accessibility and abundant stem cell content. SVF began garnering attention across diverse disciplines [19].

SVF can initiate a radical paradigm shift in understanding and addressing bone fractures, deviating away from conventional methods and searching the unexplored domains of cellular and molecular science. It integrates cellular biology and osteology, establishing novel routes for accelerated bone regeneration [21,22].

The application of SVF in treating bone fractures operates in various ways. It facilitates enhanced bone formation, wherein SVF propels the differentiation of both resident progenitor cells and those within the fraction itself into osteoblasts, thereby boosting bone formation at the fracture site. Through immune modulation, SVF, with its composition of immune cells, refines inflammatory responses, mitigating excessive inflammation and creating a favorable environment conducive to healing. SVF promotes angiogenesis, ensuring the formation of an integrated vascular network within the healing bone, which is crucial for the supply of essential nutrients and oxygen, and is crucial for optimal bone regeneration and pain reduction [23,24,25].

**Figure 1:**
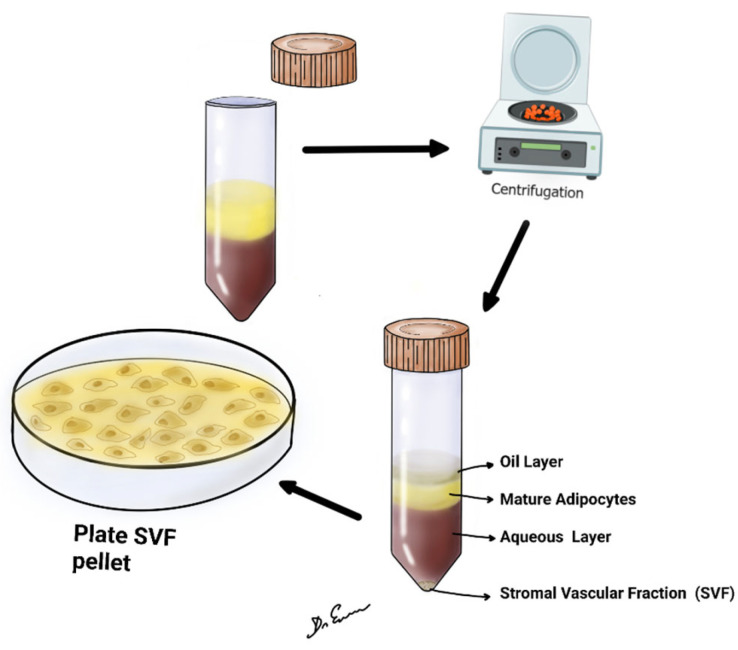
It shows lipoaspirate, centrifuged at 2500 or 3000 rpm for 4 min at room temperature. After centrifugation, upper oil fraction, middle condensed lipoaspirate, lower aqueous fraction, and the SVF were observed.

This literature review aims for a critical comparative analysis of SVF (Figure 1) with other common bone stimulation mechanisms, such as electrical stimulation, LIPUS, and BMPs. The efficacy and the potential limitations of each treatment modality in bone fracture healing are compared.

## 2. Materials and Methods

Given the extensive and detailed nature of this review, it is crucial to outline a structured and rigorous methodological framework. This would entail comprehensive literature review strategies, identification of key interventions, and an analytical approach to synthesizing gathered information. The study was registered with the International Platform of Registered Systematic Review and Meta-analysis Protocols (INPLASY). Our registration number is INPLASY2023100066.

### 2.1. Literature Search Strategy

A systematic and detailed search of the literature was conducted utilizing databases such as PubMed, Scopus, and Google Scholar. Keywords used for the search included but were not limited to “Bone Fracture Healing”, “Stroma Vascular Fraction”, “Electrical Stimulation”, “Ultrasound Stimulation”, “Mechanical Stimulation”, “Biological Interventions”, “Nutritional and Pharmacological Stimulation”, “Bone Morphogenetic Proteins”, “Platelet-Rich Plasma”, and “Stem Cell Therapy”. The literature search was confined to articles published in English from 2016 to 2023.

Inclusion Criteria: Studies included in this review met the following criteria: Peer-reviewed articles, reviews, and clinical trials focusing on SVF or other bone stimulation mechanisms in the context of bone fracture healing. Studies providing insights into the mechanisms of action, efficacy, clinical applications, and outcomes of the reviewed methods. Publications available in full text.

Exclusion Criteria: Studies not related to bone fracture healing or not focused on SVF or the compared bone stimulation mechanisms. Publications like conference abstracts, editorials, and letters, which did not provide sufficient data or detailed insights.

Data Extraction: From each selected study, the following data were extracted: the author(s), year of publication, study design, sample size, type of bone stimulation mechanism studied, clinical applications, outcomes, and limitations. A data extraction form was developed to ensure uniformity in the extraction process.

### 2.2. Quality Assessment

The quality of the included studies was rigorously assessed using appropriate critical appraisal tools. The assessment focused on the study design, methodology, result reliability, and the relevance and validity of the conclusions drawn.

Data extracted from the studies were comprehensively analyzed and integrated to draw conclusions. Comparative analyses were carried out to evaluate the mechanisms, applications, and outcomes of SVF against the other bone stimulation mechanisms. Emphasis was laid on identifying the advantages, limitations, and potential improvements of each method.

Each included study was subjected to evaluation to assess the quality of evidence presented. Studies were evaluated based on their methodological rigor, validity of findings, relevance to the review topic, and contribution to the understanding of bone fracture healing stimulation. For each selected article, data were extracted by two independent reviewers (E.N.G. and N.M.). The data comprised the year of publication, study type, number of participants (for clinical trials), main findings, and conclusions and complications. Any discrepancies between the reviewers were resolved through discussion until a consensus was reached.

Appropriate tables, graphs, and illustrations were utilized to present data and findings in a visually coherent and informative manner, aiding the reader in grasping the intricate details and complexities of each intervention.

### 2.3. Ethical Considerations

While the present review does not involve original research or direct interaction with human subjects, ethical considerations were maintained through accurate and unbiased representation of the identified studies and acknowledgement of the original sources of information.

## 3. Results

The meticulous systematic literature search initially found 39 relevant articles. However, after the diligent removal of duplicate entries and subsequent careful screening of titles and abstracts, only 22 articles were deemed fit for a more comprehensive assessment of eligibility. After the first screening, eight articles were selected, and 14 were excluded. In the last screening series of articles, five were selected. Each of these five carefully assessed articles successfully met the predetermined inclusion criteria and was therefore integrated into the review, as delineated in Table 1. The group of selected studies encompasses a diverse array of research methodologies, including prospective and/or retrospective case series, randomized controlled clinical trials, and insightful reviews.

**Figure 2:**
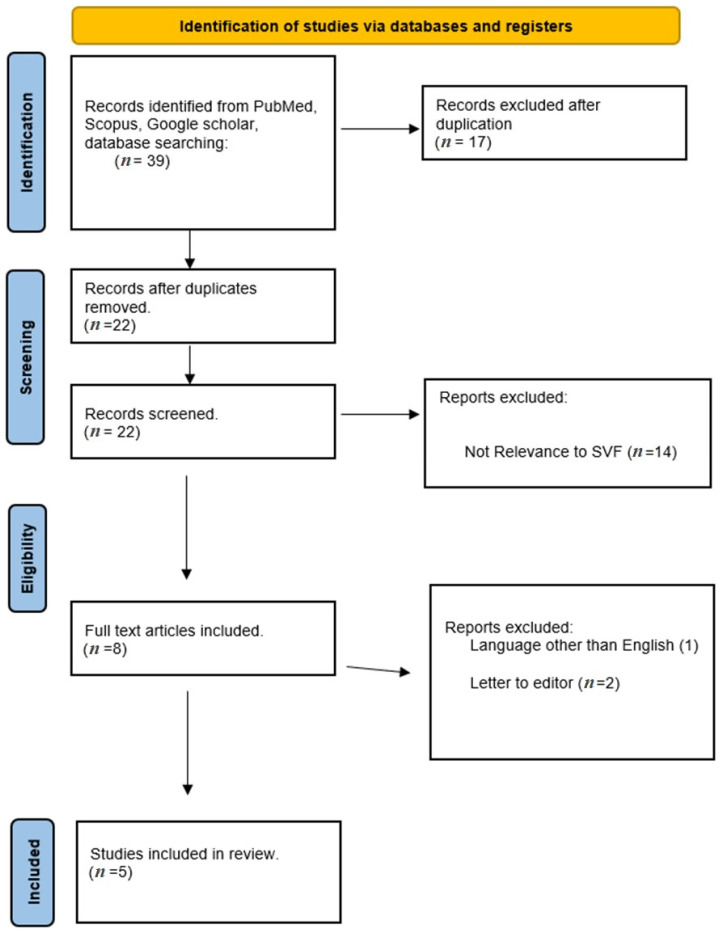
PRISMA flow diagram of this review.

This methodical and rigorous approach constructs a well-organized structure, facilitating an in-depth and systematic examination of the extant literature on SVF. This endeavor yields substantial insights, elucidating the revolutionary potential inherent in this unique cellular entity in the realm of regenerative medicine (Table 2 and Table 3). A detailed representation of the methodological progression and selection stages of the study is shown in Figure 2 through the PRISMA flow diagram.

In addition, Table 4 and Table 5 reported complications associated with SVF therapy for bone healing and complications associated with various bone healing stimulation techniques, respectively.

## 4. Discussion

**Figure 3:**
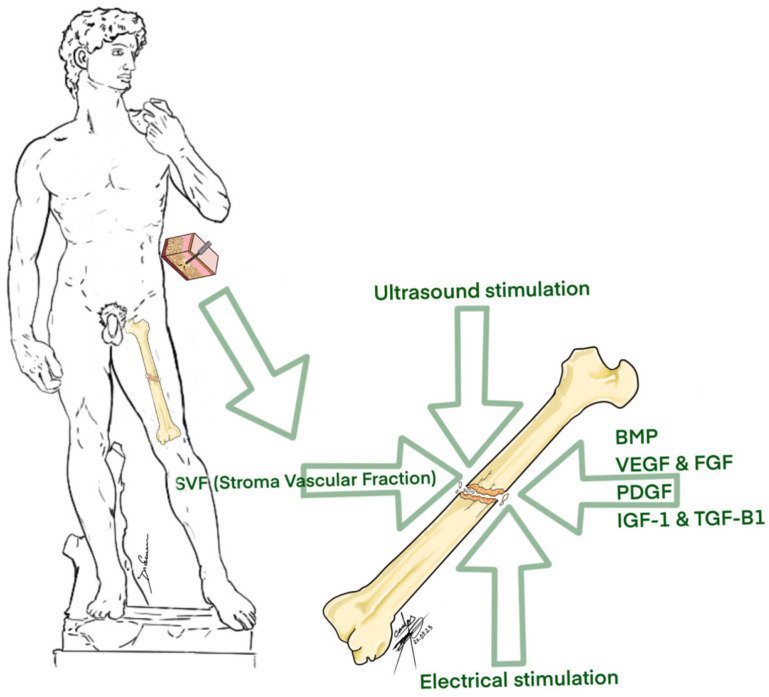
Different methods of bone fracture stimulation.

The reviewed studies collectively demonstrate the potential applications of SVF as stimulator in the bone fracture healing process (Figure 3).

The purification process ensures that unwanted components or non-functional elements are removed, leaving behind a highly enriched fraction that can be safely and effectively used for regenerative purposes [29,51,52]. The purification and analysis of SVF requires a thorough assessment of its molecular and cellular components. Cellular composition is often deciphered using flow cytometry, which uses specific markers to quantify cell types, such as ASCs (CD34+, CD31−, CD45−), endothelial cells (CD31+), and immune cells (CD45+). Additionally, microscopy, such as histological or fluorescent examinations, visually presents cellular composition (Table 6) [53], containing a diverse array of cells such as adipose-derived stem cells (ADSCs), pericytes, and smooth muscle cells. SVF has demonstrated promising regenerative, immunomodulatory, and anti-inflammatory effects (Table 6).

### 4.1. Steps of SVF

The steps of SVF separation can be summarized as (a) liposuction, (b) mechanical separation or faxination, (c) initial filtration, (d) washing, (e) final filtration, (f) SVF and adipose graft harvesting, and (g) cell counting and/or characterization (Table 7) [52,53,61,62,63,64].

In the process of finding optimal bone regeneration strategies, the exploration of SVF has presented substantial insights. Saxer et al. [26] conducted a prospective study to scrutinize the efficacy of SVF in enhancing bone regeneration, with significant improvements noted over 12 months, delineating its potential to augment bone regenerative processes effectively. Conversely, a comparison between studies like those of Urlaub et al. [27] and Sananta et al. [28] illustrated the versatility of SVF applications, from restoring bone cellularity and maturity in irradiated fractures to promoting higher expressions of TGF-β1 biomarkers, substantiating the multifaceted healing attributes of SVF in various bone defect contexts.

Differentiation from stem cell concentrators used in spinal surgery: stem cell concentrators used in spinal surgery typically involve the concentration of bone marrow aspirate [63]. This aspirate predominantly contains hematopoietic stem cells (HSCs) and a smaller proportion of mesenchymal stem cells [51,64]. In contrast, SVF derived from adipose tissue has a higher proportion of mesenchymal stem cells (MSCs) compared to HSCs [54,55]. MSCs in SVF are particularly noted for their regenerative potential in tissue engineering and regenerative medicine [65]. The extraction process for SVF from adipose tissue is different from the centrifugation and filtration methods typically used for concentrating bone marrow aspirate. The therapeutic applications of these two types of stem cell sources can differ due to their varying cellular compositions [66]. While bone marrow concentrate is often used for its potential in bone regeneration and hematopoietic support, SVF is sought after for soft tissue regeneration, immunomodulation, and angiogenesis, alongside bone healing [56,57].

The findings of Kamenaga et al. [30] illuminated the potential of both freshly isolated and cryopreserved SVF cells to enhance bone healing in non-healing fracture models, thereby broadening the scope and accessibility of SVF utilization in clinical settings. Dradjat et al. [29] also reported higher osteocalcin biomarker expressions in groups treated with SVF, indicating its promising role in bone metabolism and turnover.

These studies, while diverse in their objectives and methodologies, uniformly highlight the potential of SVF in improving various metrics of bone regeneration and vascularization compared to other stimulation methods. However, it is imperative to consider that these findings are drawn from a relatively small pool of studies with limited sample sizes, signifying the need for more extensive and diversified research to corroborate these initial observations and understand the comprehensive implications of SVF in bone healing strategies.

When comparing SVF to alternative methods such as electrical stimulation, ultrasound stimulation, and mechanical stimulation in the context of bone regeneration, it becomes evident that SVF offers several distinct advantages, despite its invasive nature. Firstly, SVF’s multifaceted therapeutic outcomes stem from its unique composition, which includes adipose-derived stem cells (ADSCs), pericytes, and smooth muscle cells. This heterogeneous mixture collaborates to create an optimal environment for healing, promoting not only bone formation but also immune modulation and vascular integration. This comprehensive approach to healing sets SVF apart from other methods, which often focus on a single aspect of bone repair [67,68]. Secondly, SVF’s ability to modulate biomarkers associated with bone regeneration, as highlighted in Table 1, showcases its potential to influence healing processes at a molecular level. This precision in targeting key factors involved in bone repair can result in superior and more predictable outcomes [69]. In comparison to other bone healing methods like electrical stimulation, ultrasound stimulation, and mechanical stimulation, SVF offers a unique advantage through its ability to address multiple facets of the healing process at the molecular level. Electrical stimulation, for instance, primarily acts through electrical fields and currents to stimulate cellular responses. Ultrasound stimulation relies on sound waves to influence osteoblast activities [6,12], while mechanical stimulation applies forces to initiate reparative responses and enhance bone density (Table 1) [10].

SVF’s versatility lies in its ability to not only activate cellular processes but also modulate molecular signals, such as the expression of key biomarkers associated with bone regeneration. The modulation of these biomarkers can lead to more predictable and superior healing outcomes [68]. For instance, the upregulation of TGF-β1 biomarkers, as seen in studies like Sananta et al. [28], signifies SVF’s role in enhancing signaling pathways critical to bone healing.

The harvesting of adipose tissue can be considered more invasive compared to the non-invasive application of electrical, ultrasound, or mechanical stimulation. This necessitates a careful evaluation of SVF’s applicability in each patient’s specific case, taking into account factors such as the extent of the fracture, the patient’s overall health, and their willingness to undergo a surgical procedure [35]. While SVF offers promising and multifaceted therapeutic outcomes for bone regeneration, its invasiveness requires a nuanced approach to patient care [35,36,70]. It should be considered as a valuable option, especially in cases where its unique capabilities align with the patient’s needs and preferences, ultimately ensuring the holistic well-being of individuals seeking bone healing interventions. Further research and clinical studies will continue to refine our understanding of SVF’s role in bone regeneration and its optimal applications [37].

The invasiveness of the procedure can lead to complications like bleeding, infection, and anesthesia-related reactions [33,34]. To mitigate these risks, advanced surgical techniques, state-of-the-art sterilization methods, and careful patient selection are employed.

The procedure also poses a risk of infection due to the potential exposure to pathogens, which is addressed through maintaining a strictly aseptic environment, using prophylactic antibiotics, and closely monitoring for post-procedure infections. Using autologous cells helps minimize rejection risks, and patients are closely monitored for adverse immune responses post-procedure. Pain and discomfort at the adipose tissue harvesting site, including swelling and bruising, are common but managed with effective pain protocols and comprehensive post-procedure care [31,54,55,56]. The risk of embolism, with fat droplets potentially entering the bloodstream, is another serious concern. This is mitigated through meticulous technique during extraction and re-injection and rigorous post-procedure monitoring. The quality and purity of SVF are also critical for therapy success, necessitating strict isolation and processing protocols and regular quality control checks.

In comparison, other bone healing stimulation techniques present different challenges. Electrical stimulation is generally low-risk but may cause skin irritation, allergic reactions to electrodes, and rare electrical burns [41]. Proper electrode placement and device maintenance are important considerations. Ultrasound stimulation carries minimal risks like skin irritation and rare thermal injuries, with careful application and intensity monitoring being crucial [43]. Mechanical stimulation varies from non-invasive to invasive, with the latter posing risks such as joint stiffness or aggravation of injury [15]. Biological intervention carries risks of immune reactions and post-surgical pain, necessitating the selection of biocompatible materials and close monitoring for rejection or inflammation. Nutritional and pharmacological stimulation is generally non-invasive but can cause hypersensitivity to supplement- and medication-specific side effects [46]. Tailoring treatment to individual dietary needs and monitoring for adverse reactions are key considerations in this approach. Each of these techniques requires a tailored approach based on the specific needs and conditions of the patient to effectively manage and mitigate the associated risks and complications.

### 4.2. Limitations of the Study

#### 4.2.1. Predominance of Alternative Methods in Literature

The conspicuous preponderance of studies exploring alternative stimulation mechanisms in the gathered literature might have skewed the comparative analysis. The disproportionate representation of electrical stimulation, LIPUS, and BMPs could have marginalized the elucidation of SVF’s unique attributes and potential contributions to bone healing.

#### 4.2.2. Scarcity of Human Studies on SVF

The lack of human clinical studies involving SVF was a salient limitation, with a predominant reliance on animal-based experimental studies. While animal models are invaluable in biomedical research, the translational efficacy of findings to human subjects remains fraught with uncertainties due to physiological, anatomical, and metabolic disparities between species. This paucity of human data limits the extrapolation and application of SVF findings to clinical human contexts, compromising the formulation of conclusive assessments and recommendations.

#### 4.2.3. Potential Publication Bias and Generalizability Concerns

The discernible scarcity of SVF studies may also be reflective of a potential publication bias, where studies with negative or inconclusive results might not have been published. This can skew the available evidence base and might lead to overly optimistic interpretations of SVF’s efficacy and applicability in bone fracture healing. Given the imbalances and disparities in the available literature, the generalizability of the comparative findings and conclusions drawn in this review is inherently circumscribed. It underscores the necessity for cautious interpretation and application of the results, particularly in the clinical translation of SVF as a viable bone stimulation method.

#### 4.2.4. Developmental Stage of SVF Research

The initial and exploratory stage of SVF research in bone fracture healing might have restricted the depth of analysis possible, in contrast to the more established and extensively studied alternative methods. The beginning stage of SVF research underlines the preliminary nature of the presented comparisons and interpretations.

Regulatory and safety concerns: As with any cell-based therapy, SVF is subject to stringent regulatory requirements to ensure safety and efficacy, which can be a barrier to its widespread adoption; regulatory agencies like the FDA have stringent requirements for cell-based therapies in bone fractures.

Technical complexity and skill requirement: The extraction, processing, and application of SVF require specialized skills and equipment, limiting its accessibility and increasing the cost.

Necessity for further research: The current developmental stage of SVF research is still in its infancy. More comprehensive and diversified studies are needed to validate preliminary observations and understand the full implications of SVF in bone healing.

## 5. Conclusions

The comparative analysis provided herein offers a novel insight into the multifaceted therapeutic potentials and limitations of SVF relative to other bone healing modalities, aiming to contribute to the body of knowledge and elucidate optimal strategies in bone regeneration and repair. However, despite the promising findings, it is crucial to acknowledge the limitations, such as the scarcity of human studies, potential publication bias, and the nascent state of SVF research. These constraints necessitate cautious interpretation and call for more comprehensive and diversified studies to validate these preliminary observations, substantiate the comparative benefits, and understand the comprehensive implications of SVF in bone healing strategies. The need for further research is critical to overcome the imbalances and disparities in the available literature, to address the generalizability concerns, and to advance the clinical translation of SVF as a viable and effective bone stimulation method. Meanwhile, the relative safety and lack of complications in reported studies position SVF as a compelling candidate in the array of bone healing modalities, offering a superior and multifaceted approach to bone regeneration and repair, subject to careful evaluation and application in suitable contexts.

## Figures and Tables

**Table 1:** Comparative analysis of articles on SVF in bone fracture healing [26,27,28,29,30].

Authors	Objectives	Methodology	Sample Size	Main Findings	Following Time (Months)
Saxer et al. [26]	To explore the efficacy of SVF in enhancing bone regeneration in fractures	Prospective	8	SVF significantly improves bone regeneration speed	12
Urlaub et al. [27]	To examine the efficacy of SVF to enhance healing outcomes in a murine model of irradiated mandibular fracture	Prospective	35	SVF therapy significantly improved all metrics of bone vascularization compared to the irradiated fracture group and was not statistically different from fracture. Bone cellularity and maturity were restored to non-irradiated levels in the irradiated fracture with SVF group despite preoperative radiation.	NA
Sananta et al. [28]	To determine the effect of SVF from adipose tissue in the process of bone defect healing, measured by TGF-β1 biomarker.	Randomized controlled trial	12	TGF- β1 biomarker expressions were higher in the group with SVF application than in the group without SVF application. All comparisons of the SVF group and positive control group showed significant differences (*p* = 0.000), respectively.	3
Dradjat et al. [29]		Randomized controlled trial	12	Osteocalcin biomarker expressions were higher in the group treated with SVF application than those without using SVF. All comparisons of the SVF group and positive control group showed significant differences (*p* < 0.05).	3
Kamenaga et al. [30]	To evaluate the therapeutic effect of (SVF) cells on fracture healing in a rat non-healing fracture model and comparing the effects between freshly isolated (F) and cryopreserved (C)-SVFs.	Prospective	5	SVF cells can enhance bone healing and cryopreserved cells have almost equal potential as fresh cells. SVF cells can be used for improving nonunion bone fracture healing as an alternative to other mesenchymal stem cells and the effect of SVF cells can be maintained under cryopreservation.	2

NA, not available.

**Table 2:** Comparative table of reviewed stimulation of bone fracture healing.

Stimulation Technique	Mechanism of Action	Application Method	Primary Benefit
Electrical stimulation [9,10]	Utilizes electric fields or currents to stimulate cellular processes involved in bone repair.	Varies depending on the type; can involve surgical insertion of electrodes or external application.	Promotes bone healing through cellular activation and biochemical reactions.
Ultrasound stimulation [10,12]	Employs sound waves of specific frequencies and intensities to influence cellular activities.	External application focusing on the fracture site.	Enhances osteoblast activities, hastening bone healing.
Mechanical stimulation [13,14]	Applies measured forces to synchronize with the body’s reparative responses.	Use of specialized devices to apply forces directly or indirectly on the fracture.	Rekindles cellular processes for bone formation and supports bone density improvement.
Biological intervention [10,19,20]	Uses natural accelerators like BMPs to speed up bone growth.	Direct application to the fracture zone.	Accelerates bone growth and regeneration.
SVF [22,23,24]	Harvested from adipose tissue; packed with cellular entities to facilitate healing.	Injection or application at the fracture site.	Enhances bone formation, immune modulation, vascular integration, and creates an optimized cellular environment for healing.
Nutritional and pharmacological stimulation [10]	Involve essential elements and medications to bolster bone’s recovery.	Oral intake or injection, as per the advised dosage.	Fortifies bones, enhances bone density, and accelerates bone repair mechanisms.

**Table 3:** Comparative overview of various bone stimulation modalities [10,11,12,13,14,15,16,17,18,19,20,21,22,23,24,25,26].

Criterion	Electrical Stimulation [11]	Ultrasound Stimulation [12]	Mechanical Stimulation [13,16]	Biological Intervention [19]	SVF [22,26]	Nutritional and Pharmacological Stimulation [20]
Invasiveness	Varies	Non-invasive	Varies	Invasive	Invasive	Non-invasive
Ease of application	Moderate	Easy	Moderate	Complex	Moderate	Easy
Targeted outcome	Bone repair	Bone formation	Bone formation/density	Bone growth/regeneration	Multi-functional	Bone fortification
Potential side effects	Minimal	Minimal	Minimal	Varies	Minimal	Minimal, dose-dependent
Applicability	Broad	Specific	Broad	Specific	Broad	Broad

**Table 4:** Complications associated with stromal vascular fraction (SVF) therapy for bone healing.

Complication	Description	Potential Impact	Mitigation Strategies
Invasiveness of procedure [31]	Surgical extraction of adipose tissue is required.	Risks like infection, bleeding, and anesthesia reactions.	Use sterile techniques; skilled surgical procedures.
Risk of infection [32,33]	Introduction of pathogens during the procedure.	Local or systemic infections.	Adhere to aseptic techniques; prophylactic antibiotics.
Immune reaction [33,34,35]	Body may react to reintroduced cells.	Inflammation, rejection, or adverse immune response.	Close monitoring; use autologous cells to reduce risk.
Pain and discomfort [36,37]	At the adipose tissue harvesting site.	Swelling, bruising, and discomfort.	Pain management; post-procedure care.
Risk of embolism [38]	Fat droplets entering the bloodstream.	Blockage in blood vessels; potentially life-threatening.	Careful handling; monitoring during and after procedure.
Quality and purity of SVF [39,40]	Isolation process must be controlled.	Reduced efficacy; introduction of other complications.	Rigorous processing protocols; quality control measures.

**Table 5:** Comparative table focusing on the complications associated with various bone healing stimulation techniques.

Stimulation Technique	Electrical Stimulation [41,42]	Ultrasound Stimulation [12,43]	Mechanical Stimulation [15,44,45]	Biological Intervention [46]	SVF (Stroma Vascular Fraction) [32,37,39,40,47]	Nutritional and Pharmacological Stimulation [48,49,50]
Invasiveness and surgical risks	Non-invasive, no surgical risks	Non-invasive, no surgical risks	May be non-invasive or invasive	Often invasive (surgical implantation)	Invasive (adipose tissue harvesting)	Non-invasive
Risk of infection	Low, at electrode sites	Low, at application site	Higher with invasive methods	High due to surgery	High due to surgery	Low, unless injections are involved
Immune and tissue reaction	Possible irritation or allergic reaction to electrodes	Minimal, possible skin irritation	Variable; higher with invasive methods	Possible immune reaction to biological materials	Potential immune reaction to reintroduced cells	Rare, mainly hypersensitivity to supplements
Pain and discomfort	Mild discomfort at application sites	Mild discomfort at application sites	Can vary; significant with invasive methods	Post-surgical pain and discomfort	Pain and swelling at harvesting site	Generally minimal
Procedure-specific complications	Skin irritation, electrical burns (rare)	Thermal injury to tissues (rare)	Joint stiffness, aggravation of injury	Rejection, inflammation, overgrowth of tissue	Embolism, variable healing efficacy, tissue damage	Side effects specific to medications or supplements
Long-term complications	Rare	Rare	Depends on method and patient response	Depends on type of biological material used	Limited long-term data available	Depends on long-term effects of medications
Need for repeat procedures	Rarely required	Rarely required	May require follow-up adjustments	May need additional treatments	Possible need for repeat procedures	Depends on treatment regimen
Efficacy and predictability	Generally predictable, efficacy varies	Efficacy can be variable	Efficacy varies widely with technique	Varies based on biological material and patient response	Unpredictable efficacy, varies by individual	Efficacy varies, dependent on condition and supplement
Other risks and considerations	Device dependency and maintenance	Inefficacy in certain cases	Dependency on device or mechanical application	Risk of over- or under-stimulation of tissue growth	Quality and purity of SVF, technique sensitivity	Nutrient imbalances, interactions with other medications

**Table 6:** SVF cell content isolated from the aqueous portion.

Type of Cells	Functions	Authors, Year [Ref.]
Mesenchymal progenitor/stem cells	Capacity to perform self-renewal, differentiation into specific cell lineages, and support maintenance of other cells via paracrine secretion.	Francis et al., 2018 [54]
Lymphocytes	Participate in both innate and adaptive immune responses with multiple effects or functions. Produce antibodies, direct cell-mediated killing of virus-infected and/or tumor cells and regulate immune responses.	Busato et al., 2020 [55]
Smooth muscle cells	Display involuntary contractile activity to control the diameter, wall movement, and wall stiffness of specific organs.	Busato et al., 2020 [55]
Adipose tissue-derived stem cells	Secrete growth factors, cytokines, and antioxidant factors into a microenvironment, regulating intracellular signaling pathways in neighboring cells. Protective outcome via inflammatory and immunomodulatory effects.	Bora et al., 2017 [22]
Preadipocytes	Promote growth of adipose tissue by differentiating into mature and metabolically active adipocytes. Proliferating preadipocytes may also exhibit phagocytic activity towards microorganisms and behave similarly to macrophage-like cells.	Matsuo et al., 2020 [56]
Mφ2 macrophage	The type 2 macrophage (Mφ2) is produced by the type 2 T helper immune response and takes on an anti-inflammatory role, typically characterized by an increase in the production of interleukins (IL-4, IL-5, IL-9 and IL-13). It is also directly involved in regenerative and tissue repair processes that occur after injuries.	Contreras et al., 2015 [57]; Dey et al., 2021 [58]
T cells	As components of the adaptive immune system with major importance, these cells are responsible for eliminating infected host cells, activating other immune cells, and secreting cytokines that further regulate immune responses.	Dulong et al., 2022 [59]
Endothelial precursor cells and endothelial cells	Differentiate into functional endothelial cells and sustain vasculo genesis by incorporating themselves into the injured endothelium with the formation of functional blood vessels and through the local secretion of pro-angiogenic factors with a paracrine effect on the cells that form the vessel. Play a critical role in vascular homeostasis as well as physiological or pathological processes such as thrombosis, inflammation, and vascular wall remodeling. Resting endothelial cells control blood flow and the passage of protein from blood into tissues, as well as inhibiting inflammation and preventing coagulation	Gulyaeva et al., 2019 [60]

**Table 7:** Steps of stromal vascular fraction separation.

	Conventional	Modified Approach
Obtaining adipose tissue	- Abdominal fat - Reusable Sorenson-type lipoaspiration cannula - Klein’s Translumination solution: modified - Klein solution (500 mL isotonic, 20 mL lidocaine, 2% epinephrine, 2 mL bicarbonate) - 50 mL Luer-Lock syringe	- Abdominal fat - Disposable/re-usable Coleman-style cannula - Klein’s Translumination solution: modified - Klein solution (500 mL isotonic, 20 mL lidocaine, 2% epinephrine, 2 mL bicarbonate) - 50 mL Luer-Lock syringe
Mechanical separation/shredding	- Shredding of tissue by shaking with glass ball (shaking time and strength depend on the user)	- Separation by the effect of gravity in a screw form mechanical separator at standard power and time
Pre-filtration	- Polyethylene filtration in a 100 μm porous polyethylene bag	- Filtration with the effect of gravity in the 100 μm porous device whose base will be supported by a metallic or polymeric cage
Washing	[–]	- Washing in the device
Final filtration	- Filtration on 10 μm porous polyethylene filters in 10 mL syringes	- Final filtration with the rise of adipose tissue and SVF to the solution surface in serum within the device
Collection of SVF/adipose tissue	- Available in an equivalent system	- Proximal adipose tissue and SVF separation reservoir
Cell counting and characterization	- Cell counting, determination of viability, determination of cell characteristics, and histochemical identification	- Cell counting, determination of viability, determination of cell characteristics, and histochemical identification

## References

[1] Bolander M.E. (1992). Regulation of fracture repair by growth factors. Proc. Soc. Exp. Biol. Med..

[2] Byock I. (2012). The Best Care Possible: A Physician’s Quest to Transform Care through the End of Life.

[3] Marsh D., Li G. (1999). The biology of fracture healing: Optimizing outcome. Br. Med. Bull..

[4] Schmidt I., Albert J., Ritthaler M., Papastavrou A., Steinmann P. (2022). Bone fracture healing within a continuum bone remodelling framework. Comput. Methods Biomech. Biomed. Eng..

[5] Marsell R., Einhorn T.A. (2011). The biology of fracture healing. Injury.

[6] Yamagiwa H., Endo N. (2009). Bone fracture and the healing mechanisms. Histological aspect of fracture healing. Primary and secondary healing. Clin. Calcium..

[7] Loi F., Córdova L.A., Pajarinen J., Lin T.H., Yao Z., Goodman S.B. (2016). Inflammation, fracture and bone repair. Bone.

[8] Veis D.J., O’Brien C.A. (2023). Osteoclasts, Master Sculptors of Bone. Annu. Rev. Pathol..

[9] Goldstein C., Sprague S., Petrisor B. (2010). Electrical Stimulation for Fracture Healing: Current Evidence. J. Orthop. Trauma.

[10] Yoshida T., Kim W.C., Kubo T. (2009). Bone fracture and the healing mechanisms. Fracture treatment using electrical stimulation. Clin. Calcium..

[11] Victoria G., Petrisor B., Drew B., Dick D. (2009). Bone stimulation for fracture healing: What’s all the fuss?. Indian J. Orthop..

[12] Watanabe Y., Matsushita T., Bhandari M., Zdero R., Schemitsch E.H. (2010). Ultrasound for fracture healing: Current evidence. J. Orthop. Trauma.

[13] Ma Q., Miri Z., Haugen H.J., Moghanian A., Loca D. (2023). Significance of mechanical loading in bone fracture healing, bone regeneration, and vascularization. J. Tissue Eng..

[14] Frost H.M. (1994). Wolff’s Law and bone’s structural adaptations to mechanical usage: An overview for clinicians. Angle Orthod..

[15] Goodman S., Aspenberg P. (1993). Effects of mechanical stimulation on the differentiation of hard tissues. Biomaterials.

[16] Komrakova M., Sehmisch S., Tezval M., Ammon J., Lieberwirth P., Sauerhoff C., Trautmann L., Wicke M., Dullin C., Stuermer K.M. (2013). Identification of a vibration regime favorable for bone healing and muscle in estrogen-deficient rats. Calcif. Tissue Int..

[17] Campos M.S., Volpon J.B., Ximenez J.P.B., Franttini A.P., Dalloul C.E., Sousa-Neto M.D., Silva R.A., Kacena M.A., Zamarioli A. (2022). Vibration therapy as an effective approach to improve bone healing in diabetic rats. Front. Endocrinol..

[18] Kanakaris N.K., Paliobeis C., Nlanidakis N., Giannoudis P.V. (2007). Biological enhancement of tibial diaphyseal aseptic non-unions: The efficacy of autologous bone grafting, BMPs and reaming by-products. Injury.

[19] Van Lieshout E.M.M., Den Hartog D. (2021). Effect of platelet-rich plasma on fracture healing. Injury.

[20] Wojda S.J., Donahue S.W. (2018). Parathyroid hormone for bone regeneration. J. Orthop. Res..

[21] Agaverdiev M., Shamsov B., Mirzoev S., Vardikyan A., Ramirez M.E., Nurmukhametov R., Beilerli A., Zhang B., Gareev I., Pavlov V. (2022). MiRNA regulated therapeutic potential of the stromal vascular fraction: Current clinical applications—A systematic review. Noncoding RNA Res..

[22] Bora P., Majumdar A.S. (2017). Adipose tissue-derived stromal vascular fraction in regenerative medicine: A brief review on biology and translation. Stem Cell Res. Ther..

[23] Montemurro N., Ortenzi V., Naccarato G.A., Perrini P. (2020). Angioleiomyoma of the knee: An uncommon cause of leg pain. A systematic review of the literature. Interdiscip. Neurosurg..

[24] Onoi Y., Matsumoto T., Sobajima S., Tsubosaka M., Hayashi S., Matsushita T., Iwaguro H., Kuroda R. (2023). Clinical use of autologous adipose-derived stromal vascular fraction cell injections for hip osteoarthritis. Regen. Ther..

[25] Kim Y.S., Oh S.M., Suh D.S., Tak D.H., Kwon Y.B., Koh Y.G. (2023). Cartilage lesion size and number of stromal vascular fraction (SVF) cells strongly influenced the SVF implantation outcomes in patients with knee osteoarthritis. J. Exp. Orthop..

[26] Saxer F., Scherberich A., Todorov A., Studer P., Miot S., Schreiner S., Güven S., Tchang L.A., Haug M., Heberer M. (2016). Implantation of Stromal Vascular Fraction Progenitors at Bone Fracture Sites: From a Rat Model to a First-in-Man Study. Stem Cells.

[27] Urlaub K.M., Ranganathan K., Lynn J.V., Luby A.O., Patrick L.N., Nelson N.S., Donneys A.M., Buchman S.R. (2021). Intraoperative Stromal Vascular Fraction Therapy Improves Histomorphometric and Vascular Outcomes in Irradiated Mandibular Fracture Repair. Plast. Reconstr. Surg..

[28] Sananta P., Dradjat R.S., Rosandi R.D., Siahaan L.D. (2022). TGF-1 biomarker level evaluation on fracture healing in a murine model with a bone defect after stromal vascular fraction application. Med. Glas.

[29] Dradjat R.S., Sananta P., Rosandi R.D., Siahaan L.D. (2021). Osteocalcin biomarker level evaluation on fracture healing with bone defect after stromal vascular fraction application in murine model. Ann. Med. Surg..

[30] Kamenaga T., Kuroda Y., Nagai K., Tsubosaka M., Takashima Y., Kikuchi K., Fujita M., Ikuta K., Anjiki K., Maeda T. (2021). Cryopreserved human adipose-derived stromal vascular fraction maintains fracture healing potential via angiogenesis and osteogenesis in an immunodeficient rat model. Stem Cell Res. Ther..

[31] Dykstra J.A., Facile T., Patrick R.J., Francis K.R., Milanovich S., Weimer J.M., Kota D.J. (2017). Concise Review: Fat and Furious: Harnessing the Full Potential of Adipose-Derived Stromal Vascular Fraction. Stem Cells Transl. Med..

[32] Zuk P.A., Zhu M., Mizuno H., Huang J., Futrell J.W., Katz A.J., Benhaim P., Lorenz H.P., Hedrick M.H. (2001). Multilineage cells from human adipose tissue: Implications for cell-based therapies. Tissue Eng..

[33] Ghigliotti G., Barisione C., Garibaldi S., Fabbi P., Brunelli C., Spallarossa P., Altieri P., Rosa G., Spinella G., Palombo D. (2014). Adipose Tissue Immune Response: Novel Triggers and Consequences for Chronic Inflammatory Conditions. Inflammation.

[34] Gornitsky J., Viezel-Mathieu A., Alnaif N., Azzi A.J., Gilardino M.S. (2019). A systematic review of the effectiveness and complications of fat grafting in the facial region. JPRAS Open.

[35] Duhoux A., Chennoufi M., Lantieri L., Hivelin M. (2013). Complications of fat grafts growth after weight gain: Report of a severe diplopia. J. Plast. Reconstr. Aesthetic Surg..

[36] Mitchell J.B., McIntosh K., Zvonic S., Garrett S., Floyd Z.E., Kloster A. (2006). Immunophenotype of human adipose derived cells: Temporal changes in stromal-associated and stem cell-associated markers. Stem Cells.

[37] Saeed K., Khan F.A., Qudus S.B.A., Javed S. (2022). Autologous Fat Grafting—A Step Forward in Wound Management. Int. J. Low. Extrem. Wounds.

[38] Kao Y., Chen K., Lee K., Hsu C., Chien Y. (2022). Pulmonary Fat Embolism following Liposuction and Fat Grafting: A Review of Published Cases. Healthcare.

[39] Debnath T., Chelluri L.K. (2019). Standardization and quality assessment for clinical grade mesenchymal stem cells from human adipose tissue. Hematol. Transfus. Cell Ther..

[40] Haack-Sørensen M., Follin B., Juhl M., Brorsen S.K., Søndergaard R.H., Kastrup J., Ekblond A. (2016). Culture expansion of adipose derived stromal cells. A closed automated Quantum Cell Expansion System compared with manual flask-based culture. J. Transl. Med..

[41] Khalifeh J.M., Zohny Z., MacEwan M., Stephen M., Johnston W., Gamble P., Zeng Y., Yan Y., Ray W.Z. (2018). Electrical Stimulation and Bone Healing: A Review of Current Technology and Clinical Applications. IEEE Rev. Biomed. Eng..

[42] Barbosa F., Garrudo F.F.F., Marques A.C., Cabral J.M.S., Morgado J., Ferreira F.C., Silva J.C. (2023). Novel Electroactive Mineralized Polyacrylonitrile/PEDOT:PSS Electrospun Nanofibers for Bone Repair Applications. Int. J. Mol. Sci..

[43] Palanisamy P., Alam M., Li S., Chow S.K., Zheng Y.P. (2022). Low-Intensity Pulsed Ultrasound Stimulation for Bone Fractures Healing: A Review. J. Ultrasound Med..

[44] Song L. (2022). Effects of Exercise or Mechanical Stimulation on Bone Development and Bone Repair. Stem Cells Int..

[45] Singh N.A., Quine S., Clemson L.M., Williams E.J., Williamson D.A., Stavrinos T.M., Grady J.N., Perry T.J., Lloyd B.D., Smith E.U.R. (2012). Effects of high-intensity progressive resistance training and targeted multidisciplinary treatment of frailty on mortality and nursing home admissions after hip fracture: A randomized controlled trial. J. Am. Med. Dir. Assoc..

[46] Massari L., Benazzo F., Falez F., Perugia D., Pietrogrande L., Setti S., Osti R., Vaienti E., Ruosi C., Cadossi R. (2019). Biophysical stimulation of bone and cartilage: State of the art and future perspectives. Int. Orthop..

[47] Wu V., Bravenboer N., Ten Bruggenkate C.M., Helder M.N., Schulten E.A. (2023). Long-Term Safety of Bone Regeneration Using Autologous Stromal Vascular Fraction and Calcium Phosphate Ceramics: A 10-Year Prospective Cohort Study. Stem Cells Transl. Med..

[48] Tarantino U., Cerocchi I., Celi M., Scialdoni A., Saturnino L., Gasbarra E. (2009). Pharmacological agents and bone healing. Clin. Cases Miner. Bone Metab..

[49] AI-Aql Z.S., Alagl A.S., Graves D.T., Gerstenfeld L.C., Einhorn T.A. (2008). Molecular mechanisms controlling bone formation during fracture healing and distraction osteogenesis. J. Dent. Res..

[50] Pountos I., Georgouli T., Blokhuis T.J., Pape H.C., Giannoudis P.V. (2008). Pharmacological agents and impairment of fracture healing: What is the evidence?. Injury.

[51] Karamian B.A., Schroeder G.D., Lambrechts M.J., Canseco J.A., Oner C., Vialle E., Rajasekaran S., Dvorak M.R., Benneker L.M., Kandziora F. (2023). An international validation of the AO spine subaxial injury classification system. Eur. Spine J..

[52] Copcu H.E., Oztan S. (2021). Not Stromal Vascular Fraction (SVF) or Nanofat, but Total Stromal-Cells (TOST): A New Definition. Systemic Review of Mechanical Stromal-Cell Extraction Techniques. Tissue Eng. Regen. Med..

[53] Pak J., Lee J.H., Pak N.J., Park K.S., Jeon J.H., Jeong B.C., Lee S.H. (2018). Clinical Protocol of Producing Adipose Tissue-Derived Stromal Vascular Fraction for Potential Cartilage Regeneration. J. Vis. Exp..

[54] Francis S.L., Duchi S., Onofrillo C., Di Bella C., Choong P.F.M. (2018). Adipose-Derived Mesenchymal Stem Cells in the Use of Cartilage Tissue Engineering: The Need for a Rapid Isolation Procedure. Stem Cells Int..

[55] Busato A., De Francesco F., Biswas R., Mannucci S., Conti G., Fracasso G., Conti A., Riccio V., Riccio M., Sbarbati A. (2020). Simple and Rapid Non-Enzymatic Procedure Allows the Isolation of Structurally Preserved Connective Tissue Micro-Fragments Enriched with SVF. Cells.

[56] Matsuo F.S., Cavalcanti de Araújo P.H., Mota R.F., Carvalho A.J.R., de Queiroz M.S., de Almeida B.B., Ferreira K.C.d.O.S., Metzner R.J.M., Ferrari G.D., Alberici L.C. (2020). RANKL induces beige adipocyte differentiation in preadipocytes. Am. J. Physiol. Endocrinol. Metab..

[57] Contreras G.A., Kabara E., Brester J., Neuder L., Kiupel M. (2015). Macrophage infiltration in the omental and subcutaneous adipose tissues of dairy cows with displaced abomasum. J. Dairy Sci..

[58] Dey A., Ni Z., Johnson M.S., Sedger L.M. (2021). A multi-colour confocal microscopy method for identifying and enumerating macrophage subtypes and adherent cells in the stromal vascular fraction of human adipose. J. Immunol. Methods.

[59] Dulong J., Loisel S., Rossille D., Léonard S., Bescher N., Bezier I., Latour M., Monvoisin C., Monnier D., Bertheuil N. (2022). CD40L-expressing CD4+ T cells prime adipose-derived stromal cells to produce inflammatory chemokines. Cytotherapy.

[60] Gulyaeva O., Dempersmier J., Sul H.S. (2019). Genetic and epigenetic control of adipose development. Biochim. Biophys. Acta Mol. Cell Biol. Lipids.

[61] Zhu H., Ge J., Chen X., Lu F., Cai J. (2019). Mechanical Micronization of Lipoaspirates for Regenerative Therapy. J. Vis. Exp..

[62] Goncharov E.N., Koval O.A., Nikolaevich Bezuglov E., Ramirez M.d.J.E., Engelgard M., Igorevich E.I., Saporiti A., Kotenko K.V., Montemurro N. (2023). Stromal Vascular Fraction Therapy for Knee Osteoarthritis: A Systematic Review. Medicina.

[63] Goncharov E.N., Koval O.A., Igorevich E.I., Encarnacion Ramirez M.D.J., Nurmukhametov R., Valentinovich K.K., Montemurro N. (2024). Analyzing the Clinical Potential of Stromal Vascular Fraction: A Comprehensive Literature Review. Medicina.

[64] Hachem L.D., Ahuja C.S., Fehlings M.G. (2017). Assessment and management of acute spinal cord injury: From point of injury to rehabilitation. J. Spinal Cord Med..

[65] Montemurro N., Murrone D., Romanelli B., Ierardi A. (2020). Postoperative Textiloma Mimicking Intracranial Rebleeding in a Patient with Spontaneous Hemorrhage: Case Report and Review of the Literature. Case Rep. Neurol..

[66] Viola A., Appiah J., Donnally C.J., Kim Y.H., Shenoy K. (2022). Bone Graft Options in Spinal Fusion: A Review of Current Options and the Use of Mesenchymal Cellular Bone Matrices. World Neurosurg..

[67] Montemurro N., Pierozzi E., Inchingolo A.M., Pahwa B., De Carlo A., Palermo A., Scarola R., Dipalma G., Corsalini M., Inchingolo A.D. (2023). New biograft solution, growth factors and bone regenerative approaches in neurosurgery, dentistry, and orthopedics: A review. Eur. Rev. Med. Pharmacol. Sci..

[68] Bhattacharjee M., Escobar Ivirico J.L., Kan H.M., Shah S., Otsuka T., Bordett R., Barajaa M., Nagiah N., Pandey R., Nair L.S. (2022). Injectable amnion hydrogel-mediated delivery of adipose-derived stem cells for osteoarthritis treatment. Proc. Natl. Acad. Sci. USA.

[69] Navarro A., Marín S., Riol N., Carbonell-Uberos F., Miñana M.D. (2015). Fibroblast-Negative CD34-Negative Cells from Human Adipose Tissue Contain Mesodermal Precursors for Endothelial and Mesenchymal Cells. Stem Cells Dev..

[70] Montemurro N., Cocciaro A., Liberti G., Cosottini M., Perrini P. (2022). The internal trabecular bone structure of the odontoid process of the axis. A retrospective single-center comparative study in patients following cervical trauma. J. Neurol. Surg. A Cent. Eur. Neurosurg..

